# Chemical Composition and Cytotoxic and Antibacterial Activities of the Essential Oil of* Aloysia citriodora* Palau Grown in Morocco

**DOI:** 10.1155/2017/7801924

**Published:** 2017-06-12

**Authors:** Moulay Ali Oukerrou, Mounir Tilaoui, Hassan Ait Mouse, Inass Leouifoudi, Abdeslam Jaafari, Abdelmajid Zyad

**Affiliations:** Laboratory of Biological Engineering, Natural Substances, Cellular and Molecular Immuno-Pharmacology, Immunobiology of Cancer Cells Cluster, Faculty of Science and Technology, Sultan Moulay Slimane University, Beni Mellal, Morocco

## Abstract

The aim of this work is to investigate the* in vitro* cytotoxic and antibacterial effects of the essential oils of* Aloysia citriodora* Palau, harvested in different regions of Morocco. The chemical profile was established using gas chromatography-mass spectrometry analysis. The cytotoxic activity against P815, MCF7, and VERO cell lines as well as the normal human peripheral blood mononuclear cells (PBMCs) was evaluated using the MTT assay. Standard, ATCC, strains of bacteria (*Escherichia coli*,* Staphylococcus aureus*, and* Pseudomonas aeruginosa*) were cultivated in Muller Hinton media. Then, agar disc diffusion, minimum inhibitory concentrations (MICs), and minimal bactericidal concentrations (MBCs) were determined using microdilution method. The essential oils obtained were predominantly composed of *β*-spathulenol (15.61%), Ar-curcumene (14.15%), trans-caryophyllene oxide (14.14%), and neral (10.02%). The results of the assays showed that the cytotoxic effect of the essential oil of* A. citriodora* was high on P815 and moderate on MCF7 and on VERO cell lines. However, no cytotoxic effect was observed on PBMCs. On the other hand, essential oils showed a significant antimicrobial activity against both Gram-negative and Gram-positive bacteria. MICs ranged between 2.84 and 8.37 mg/ml. Essential oil of* A. citriodora* leaves possesses significant antibacterial effect and cytotoxic activity against tumor cell lines.

## 1. Introduction

The genus* Aloysia* belongs to the Verbenaceae family and consists of approximately 200 species of herbs, shrubs, and small trees which are often aromatic [1]. The species* Aloysia citriodora* Palau or* Lippia citriodora* (H.B. & K.) is commonly known as Lemon Verbena, Verbena grass Louise, Arabic tea, and lemongrass. This plant is growing spontaneously in South America, especially in Argentina and Chile. In Morocco, this species is cultivated for more than a century and has been used in folk medicine as herbal tea preparations, for its antispasmodic, digestive, stomachic, sedative, and antipyretic properties. The essential oil extracted from the dried leaves of* A. citriodora* is indicated for anxiety, stress, insomnia, some depressions, nervous fatigue, multiple sclerosis, psoriasis, tachycardia, rheumatism, enterocolitis, Crohn's disease, anorexia, dyspepsia, intestinal parasites (amebiasis and amebic cysts), and prevention of asthma attacks [2–4]. The broad range of biological activities of essential oils could be generally correlated to the chemical composition. Therefore, this biological difference can be partly explained by the variation in their chemical composition [5]. It is well established that sesquiterpenoids and their derivatives are credited with many biological activities such as anti-inflammatory, antibacterial, antiasthmatic, and antifungal properties [6]. Thus, structure-activity relationships describe broad classes of activities for the different chemical groups of molecules found in essential oils.

In the present study, we analyzed the chemical composition analysis; the cytotoxic and the antibacterial activities of essential oil of* A. citriodora* distilled from the shade-dried leaves harvested in the summer season (July-August 2015) in different Moroccan regions.

## 2. Material and Methods

### 2.1. Plant Material

The leaves of* A. citriodora* Palau were collected during July-August 2015 (the period of maximum essential oils production) [7], from the following Moroccan regions: Marrakech (Ait Imour region), Beni Mellal (Laâyayta locality), Agadir (Oulad Tayma), and Berkane and Demnate (mountain region). The plants were grown in organic farming without pesticide treatments. The botanical determination was performed by Pr. A. Boulli (Laboratory of Natural Resources Valorization, Faculty of Science and Technology, Beni Mellal), and a voucher specimen was deposited in the Herbarium at the Faculty of Science and Technology, Beni Mellal, Morocco, under reference: FSTBMCOLL72015.

### 2.2. Extraction of the Essential Oil

The collected leaves of* A. citriodora* Palau were shade-dried during two weeks. Then the essential oil was obtained by hydrodistillation using a Clevenger-type device (samples were 100 g of crushed leaves in 1.5 l distilled water). At the end of the distillation process, the organic phase which contains the essential oil is separated from the hydrosol (aqueous phase) with ether followed by gentle removal of the solvent by evaporation. The yield of essential oil ranged from 0.1% to 0.2%.

### 2.3. GC-MS

The obtained essential oil samples were analyzed with a gas chromatograph Trace GC Ultra equipped with a FID detector and a capillary column (30 m × 0.25 mm, 0.25 *μ*m film thickness) DB-5 (methyl polysiloxane with 5% phenyl); the injection volume was 1 *μ*L. The gas chromatograph was coupled to a mass spectrometer Q Polaris MS that performs the analysis of the mass spectrometry (70 eV with an ion trap). The temperature program was 40°C for 2 min and then heated to 180°C at a rate of 4°C/min. The carrier gas was helium (1.4 ml/min).

### 2.4. Cytotoxicity Assay

The cytotoxic effect of essential oils was evaluated against P815 (Murine Mastocytoma, cell line), MCF7 (human breast adenocarcinoma), and VERO (kidney carcinoma cell line of monkey). The used cell lines were obtained as a gift of Professor G. Lemaire, Institute of Biochemistry, University of Paris XI, France. PBMCs were isolated using the standard Ficoll-Hypaque density gradient from samples of healthy volunteers under medical supervision. These cells were maintained and grown in special culture medium (RPMI-1640, Sigma-Aldrich, France) supplemented with 10% heat inactivated fetal calf serum (FCS) and 1% penicillin-streptomycin (Sigma-Aldrich). Cultures were maintained at 37°C in humidified 5% CO_2_ incubator. The cytotoxicity assays were performed in triplicate on each cell line using the MTT [3-(4,5-dimethylthiazol-2-yl)-2,5-diphenyltetrazolium bromide] assay (Sigma-Aldrich), as described and modified by Mosmann [8] and Mouse et al. [9].

All experiments were performed with twice PBS washed cells at a density of about 1.5 × 10^4^ cells per well (1.5 × 10^5^ cells/ml), in flat-bottomed 96-well microtiter plates (Thermo Fisher Scientific) in 100 *μ*l complete medium per well. Then 100 *μ*l of complete culture medium containing different concentrations of the tested essential oils (dissolved in DMSO) was added to each well. After 48 h exposure of cells to these concentrations of tested essential oils at 37°C and 5% CO_2_, 100 *μ*l of medium was carefully aspirated from each well and replaced with 20 *μ*l of MTT solution (5 mg/ml of PBS). After incubation in the same conditions for 4 hours, the plates were treated with 80 *μ*l of an HCl/isopropanol (24 : 1) solution to dissolve the blue intracellular formazan produced by living cells. One hour later, the absorbance (optical density OD) of the plates was determined by a Multiskan EX spectrophotometer reader at two wavelengths (540 and 630 nm). DMSO (0.5%) was used as the negative control and methotrexate (MTX) as the positive one.

The viability was evaluated by the following formula:

(1) $$\begin{array}{c} \%\;\;Viability=\left(\;\frac{OD}{{OD}_{0}}\;\right)\times 100. \end{array}$$

OD is the optical density of the solution in wells containing cells treated with essential oils and OD_0_ is the optical density of the solution in wells containing DMSO treated cells (negative control). The test was performed in triplicate. The IC_50_ values were graphically determined using the Microsoft excel 2013 edition.

### 2.5. Bacterial Culturing Conditions

The bacterial strains (*E. coli* ATCC 25922,* S. aureus* ATCC 25923,* and P. aeruginosa* ATCC 27853) have been kindly provided by the Laboratory of Bioprocess and Biointerface of the Faculty of Sciences and Technology of Beni Mellal, Morocco. These bacteria were maintained by subculture on Mueller-Hinton nutrient agar medium.

#### 2.5.1. Disc Diffusion Assay

The determination of inhibition diameters of essential oils on* E. coli* ATCC 25922,* S. aureus* ATCC 25923, and* P. aeruginosa* ATCC 27853 cultures was carried out (in triplicate) by the agar disc diffusion method according to the NCCLS (the National Committee for Clinical Laboratory Standards), recently called CLSI (Clinical Laboratory Standards Institute) [10]. The Mueller-Hinton agar media in Petri dishes was swabbed with 100 *μ*l of the bacterial suspension 10^8^ CFU/ml (0.5 NTU McFarland) and kept for 30 min at 4°C. Sterile filter paper discs (6 mm in diameter) [11] were soaked with 6 *μ*l essential oil and placed aseptically on the surface of the inoculated Muller-Hinton agar plates. Plates were then incubated at 37°C for 24 h and the diameters of bacterial growth inhibition zones (in millimeters) were recorded. Standard antibiotic discs (6 mm) were used as positive controls: ceftriaxone (30 *μ*g), ofloxacin (5 *μ*g), and blank discs were used as negative control.

#### 2.5.2. MICs and MBCs Determination

The MIC (minimal inhibitory concentration) and MBC (minimal bactericidal concentration) tests were performed (in triplicate) by the broth microdilution method. The essential oils were dissolved in DMSO (Sigma). Serial dilutions (1 : 2) from 2.5% (v/v) as the essential oil concentrations to be incubated at 37°C for 24 h with the bacterial suspensions adjusted to 10^5^ CFU/ml in wells of 96-well microplates (Thermo Fisher Scientific). Untreated control (wells containing bacteria with DMSO at a concentration of 1%) was included in the assay.

After incubation, the wells were optical density examined (OD_600_ nm) with a spectrophotometer (Multiskan EX) for bacterial growth.

The MIC was determined as follows:

(2) $$\begin{array}{c} MIC=\left[\;\frac{\left({\text{OD}}_{Untreated\;\;control}-{\text{OD}}_{essential\;\;oils}\right)}{{\text{OD}}_{Untreated\;\;control}}\;\right]\times 100. \end{array}$$

OD_essential  oils_ is the optical density of the suspension in wells containing essential oils treated bacteria and OD_Untreated  control_ is the optical density of the suspension in wells containing DMSO (without essential oils) treated bacteria (negative control).

The MIC is defined as the lowest concentration of the essential oil at which the bacteria does not show visible growth.

MBC is defined as the lowest concentration of the essential oil at which incubated microorganisms were completely killed.

### 2.6. Statistical Analysis

Data were expressed as mean values ± SEM of three different experiments; each one was performed in duplicate. Statistical significance was determined with the one-way analysis of variance followed by a post hoc Scheffe's test. The differences were considered statistically significant at *p* < 0.05.

## 3. Results

### 3.1. Extraction and Chemical Composition of the Essential Oils

Distilled essential oil of* A. citriodora* features: appearance (mobile liquid, clear), color (yellow, more or less dark), characteristic odor (lemony, fresh), and density at 20°C (0.879).

**Figure 1:**
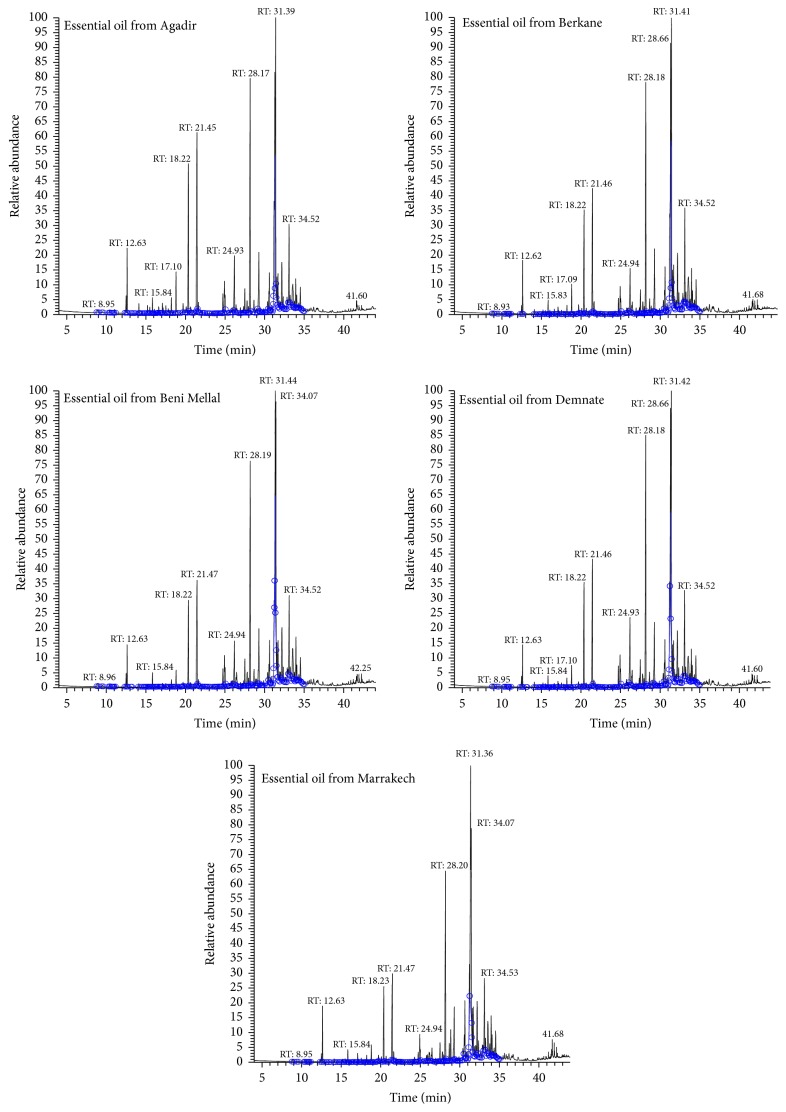
Chromatograms of* A. citriodora* essential oils from different regions.

Among 72 peaks revealed by chromatography (Figure 1), 64 peaks (96%) were identified (according to National Institute of Standards and Technology database). Twenty-four among 64 peaks represent 80% of the essential oil compounds (Table 1). Eight peaks were for unknown compounds and probably represent 4% of the essential oils.

Molecules belonging to five biochemical families, namely, sesquiterpenes (Ar-curcumene 12.32%), terpene oxides (caryophyllene oxide 13.68%), sesquiterpenols (spathulenol 12.38%), monoterpene aldehydes (neral 8.07%), and monoterpenols (cis-verbenol 6.28%), constitute 53% of the essential oil's major compounds (Table 2).

### 3.2. Essential Oils Mediated Cell Cytotoxicity

**Figure 2:**
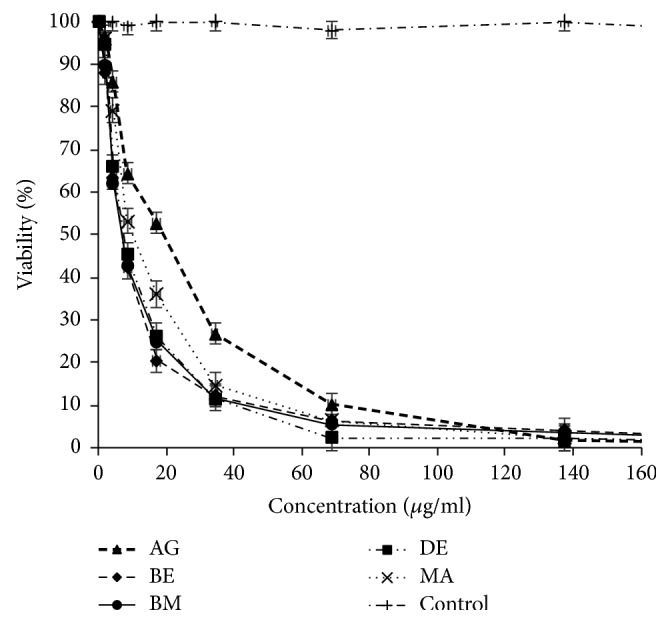
*A. citriodora* essential oil's cytotoxicity against P815 cell line. AG: Agadir. BM: Beni Mellal. BE: Berkane. DE: Demnate. MA: Marrakech. DMSO: control.

**Figure 3:**
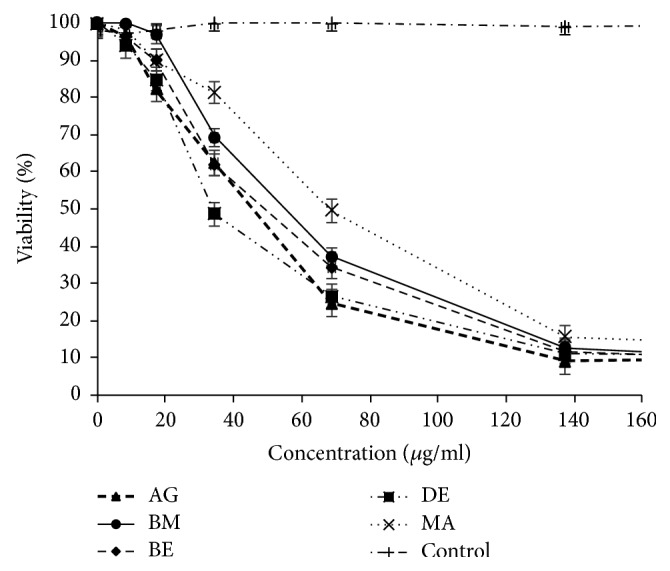
*A. citriodora* essential oil's cytotoxicity against MCF7 cell line. AG: Agadir. BM: Beni Mellal. BE: Berkane. DE: Demnate. MA: Marrakech. DMSO: control.

**Figure 4:**
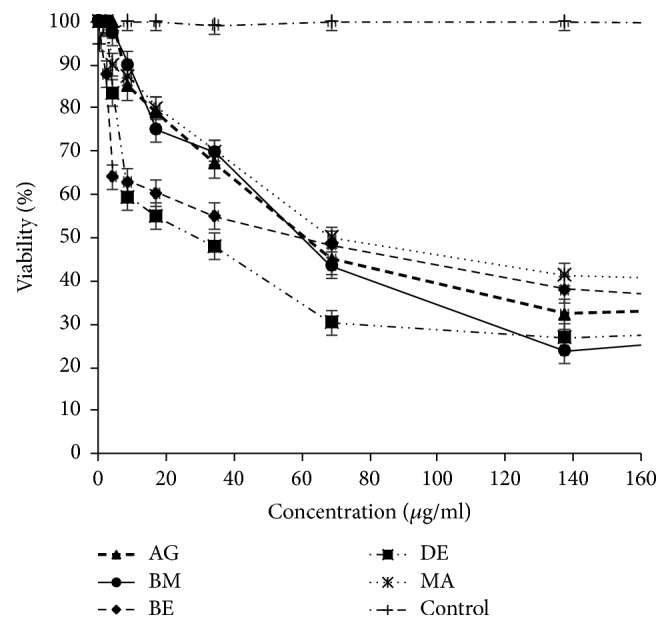
*A. citriodora* essential oil's cytotoxicity against VERO cell line. AG: Agadir. BM: Beni Mellal. BE: Berkane. DE: Demnate. MA: Marrakech. DMSO: control.

**Figure 5:**
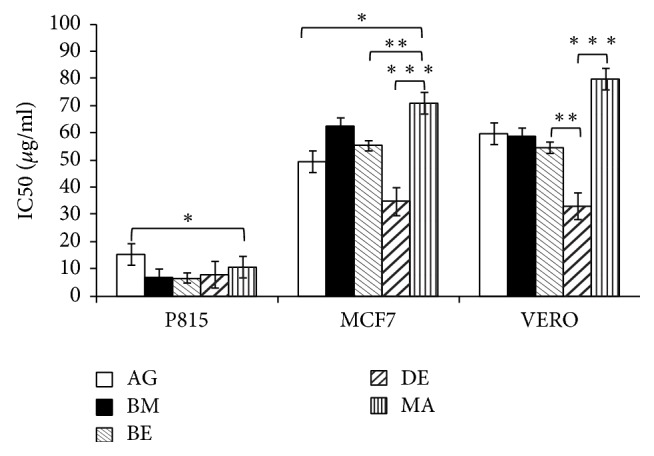
IC_50_ of essential oils of* A. citriodora* against P815, MCF7, and VERO cell lines. AG: Agadir, BM: Beni Mellal, BE: Berkane, DE: Demnate, and MA: Marrakech. The data shown are the mean values ± SEM of three different experiments; each one was performed in duplicate. Statistical significance was determined with the one-way analysis of variance followed by a post hoc Scheffe's test. Significance was considered at *p* < 0.05 (*∗*), *p* < 0.01 (*∗∗*), and *p* < 0.001 (*∗∗∗*) compared to control (untreated cell).

**Figure 6:**
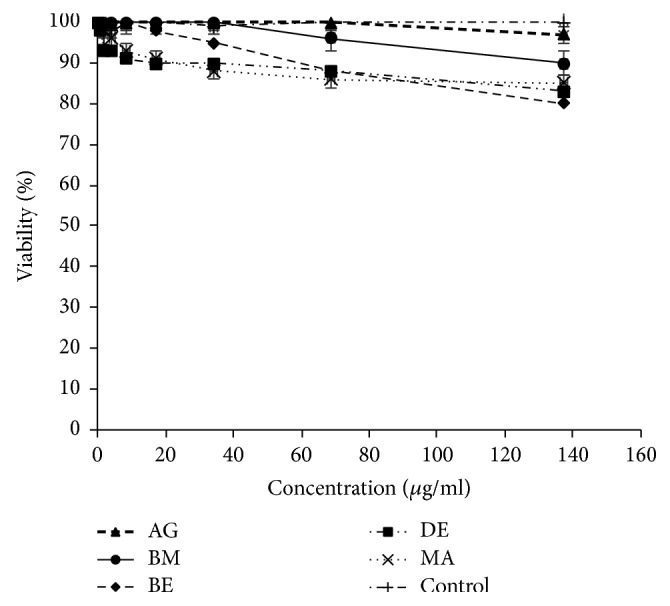
Cytotoxicity assay of* A. citriodora* essential oils from different Moroccan regions on PBMCs. AG: Agadir. BM: Beni Mellal. BE: Berkane. DE: Demnate. MA: Marrakech. DMSO: control.

The assays showed that the essential oil of* A. citriodora* exerted a dose dependent cytotoxic effect on P815, MCF7, and VERO tumor cell lines with IC_50_ ranging from 6.60 to 79.63 *μ*g/ml. (Figures 2, 3, 4, and 5). However, no cytotoxicity was observed against PBMCs. In fact, the viability was over 80% at the concentration used to induce tumor cell lysis (Figure 6).

### 3.3. Antibacterial Assays

#### 3.3.1. Disc Diffusion Assay

Inhibition zone diameters of the disc diffusion assays were recorded in Table 3. The results indicate that* E. coli* ATCC 25922 and* S. aureus* ATCC 25923 were sensitive to essential oils of* A. citriodora,* while* P. aeruginosa* ATCC 27583 was resistant.

#### 3.3.2. Determination of MIC and MBC

The obtained values of MIC and MBC are shown in Table 4. The MIC on* E. coli* ranged from 2.84 to 3.73 mg/ml for MA and BM essential oils, respectively.* S. aureus* MICs were 3.51 mg/mL for BM and 3.87 mg/ml for DE essential oils.

## 4. Discussion

### 4.1. Chemical Composition Analysis

The chromatographic spectra of different essential oils from different Moroccan regions revealed the same qualitative chemical composition with some quantitative differences. *β*-spathulenol is the most abundant compound and represents 15% of the essential oil from Berkane region. trans-Caryophyllene oxide prevails in essential oils from all regions with an average rate of 14%. Ar-curcumene is found to represent 14% of the essential oils from Beni Mellal and Agadir regions and 11% of those from other regions. Neral represents 10% of the essential oil from Agadir region and 6% to 8% of those from other regions. cis-Verbenol represents nearly 8% of the essential oil from Agadir region and 4% to 6% of other essential oils. Paracymene was only present in the essential oil from Agadir region at 0.02%. *α*-muurolene was only found at 0.04% in the essential oils from Beni Mellal and Demnate regions. 8-Cedren-13-ol was not found in the essential oil from Agadir and represented 0.01 to 0.05% of the essential oils from other regions.

The major molecules dominating the chemical composition of essential oils are caryophyllene oxide, *β*-spathulenol, Ar-curcumene, neral, and cis-verbenol (Table 2). Other products such as *β*-caryophyllene, isoledene, elemene, 1.8-cineole, copaene, and nerol are also present with variable ratios. Unlikely, limonene and 6-methyl-5-hepten-2-one, which are compounds constantly found in* A. citriodora* essential oils distilled in Morocco at proportions of 14% for the first and 3% for the second, are present in the studied essential oils in the present work at very low averaging rates of 0.02% to 0.5%. In fact, the compositions of the essential oils depend on the operating (drying, extraction methods) and on the harvest and the storage conditions [12, 13].

The chemical composition analysis of our essential oil was dominated mainly by Ar-curcumene (12.32%), caryophyllene oxide (13.68%), and spathulenol (12.38%). Other compounds were reported in the essential oil of Argentina which is rich in ketones (myrcenone 36.50%, *α*-thujone 13.10%), while limonene is only at 6.87% [14]. The essential oil from Turkey contains citrals ranging from 17.90% to 27.10% and limonene of 16% [15]. The major compounds of the essential oil of* A. citriodora* from Portugal were geranial (26.80% to 38.30%), neral (20.80% to 29.60%), and limonene (5.70% to 20.60%) [13].

The main compounds found in the present investigation were different quantitatively and qualitatively from those reported in the literature. The average rate of limonene and geranial was 17% and 9%, respectively, in different essential oils distilled from* A. citriodora* grown in Morocco (leaves of* A. citriodora* harvested in May period), whereas the amount of the monoterpene (limonene) is only 0.52% and the terpene aldehyde (geranial) is not detected in the present study. However, the rates of sesquiterpenes (*β*-caryophyllene, Ar-curcumene, *β*-elemene, etc.), terpene oxides (caryophyllene oxide), some monoterpene alcohols (cis-verbenol), and sesquiterpene alcohols (spathulenol) were higher in the present studied essential oils [16]. It is interesting to note that limonene, *β*-caryophyllene, p-cymene, linalool, citral, *α*-pinene, and 1.8-cineole are common compounds of essential oils of* Lippia* sp. and thought to be responsible for specific biological effects and properties attributed to the genus* Aloysia* [4].

### 4.2. Cytotoxicity against P815, MCF7, VERO, and PBMCs Cell Lines

Based on the essential oils cytotoxicity ranking [17], the five essential oils studied marked very high cytotoxic effect against P815 (BE essential oil is the most cytotoxic with IC_50_ = 6.60 *μ*g/ml) and high to moderate activity against MCF7 and VERO cell lines (DE essential oil is the most cytotoxic with IC_50_ = 34.72 *μ*g/ml and 32.90 *μ*g/ml, resp.). To the best of our knowledge, there is no previous report about the cytotoxic activity of* A. citriodora* essential oil. However, the cytotoxic effect of* Lippia alba*, with its main constituent citral on HeLa and VERO cell lines was reported [18]. The interesting cytotoxicity toward cell lines could be due to the main compounds in the essential oil. Literature reports the induction of apoptosis by citrals (neral and geranial) in chronic lymphoid leukemia by activation of caspase-3 [19, 20]. Moreover, it has also been demonstrated that *β*-caryophyllene and caryophyllene oxide were also reported to induce apoptosis in tumor cells and exert analgesic, anti-inflammatory, antibacterial, antifungal, and sedative activities [21, 22]. Furthermore, *β*-elemene and spathulenol were reported as having anticancer activities on human glioblastoma and on MCF7 cancer cell lines [23–25]. Interestingly, no cytotoxic effect of* A. citriodora* essential oil studied here was observed on Human peripheral blood mononuclear cells (PBMCs).

### 4.3. Antibacterial Effect

The MIC values (Table 4) showed that the MA and BM essential oils were the most active against* E. coli* and BM and DE were the most active against* S. aureus*. In contrast, AG and BE were the least effective on* E. coli* and on* S. aureus*, respectively. All the five tested essential oils showed no activity against* P. aeruginosa*. Other studies have shown that this bacterial strain is naturally resistant to other essential oils. The CMB/MIC ratio (2.14 for* E. coli* and 2.02 for* S. aureus*) permitted to qualify the five studied essential oils as bactericidal [26]. Interestingly, it was observable that cytotoxic and antibacterial activities of essential oils were different; for example, MA presented the moderate cytotoxicity but a very strong antibacterial activity. This may be explained by qualitative and quantitative variability of chemical compounds identified in these essential oils and their differential molecular mechanisms.

Generally, the first site of action of essential oils on the bacterial cells is the membranes. This is directly related to the hydrophobicity of the essential oils components. This property ensures and facilitates permeability of membrane phospholipids bilayer to these molecules. The result is a destabilization of the plasma membrane structure and a change in its permeability to ions, protons, and other cellular components [27–30]. In addition to the induced membrane alterations, such molecules can cross the lipid bilayer and interact with intracytoplasmic targets [31]. Given the molecular diversity of the essential oils, it appears more likely that their antibacterial activity results from the combination of several mechanisms, acting synergistically on different cellular targets [32]. In fact, among the dominant molecules in the five studied essential oils, caryophyllene and *β*-caryophyllene oxide have antibacterial and antifungal activities [33]. Oxygenates (caryophyllene oxide, 1.8-cineole, etc.) have* in vitro* antibacterial activity [34]; citral (neral and geranial) has a strong antibacterial effect [35]. The interaction between p-cymene, ‎*γ*-terpinene, and the phenolic compounds could exhibt an antibacterial activity [36]. Furthermore, the essential oils showing high antibacterial activity are phenol and cinnamic aldehyde rich oils (thymol, carvacrol, and eugenol) as in the essential oils of* Thymus vulgaris*,* Origanum compactum*,* Satureja montana*,* Eugenia caryophyllata*, and* Cinnamomum zeylanicum* [37]. However, antimicrobial effectiveness of an essential oil is due to the nature and content of these various constituents that may act synergistically; the effect of minor compounds is not always negligible. Synergism between constituents can cause a much more pronounced effect than the expected activity of the major compounds [38].

## 5. Conclusion

The results obtained in this study showed that the molecular profile of the essential oil of* A. citriodora* grown in Morocco is slightly variable depending on the region where the plant was harvested.* In vitro* tested essential oils of* A. citriodora* have a strong cytotoxic activity against P815 (IC_50_ = 6.60 *μ*g/ml), compared to MCF7 (IC_50_ = 34.72 *μ*g/ml) and VERO (IC_50_ = 32.90 *μ*g/ml). A moderate antibacterial activity on* E. coli* ATCC 25922 and on* S. aureus* ATCC 25923 was recorded. However,* P. aeruginosa* ATCC 27853 strain was resistant to these essential oils. Cytotoxic and antibacterial activities depend on the chemical nature and interactions of* A. citriodora* essential oil's compounds. Further studies need to be conducted on apoptosis induction, genes expression (Bcl2, p53, Bax, Jun/Fos, etc.), and antitumor activity of the main compounds of* A. citriodora* essential oil to understand the involved molecular pathways.

## Figures and Tables

**Table 1:** Chemical composition after GC-MS analysis of the essential oils of *A. citriodora*.

Peak number	Component^a^	RI^b^	% AG^c^	% BM^d^	% BE^e^	% DE^f^	% MA^g^	Identification
1	*α*-Pinene	909	0.23	0.39	0.17	0.21	0.20	RI, MS
2	*α*-Thujene	927	0.09	0.14	0.08	0.09	0.08	RI, MS
3	*β*-Pinene	964	0.14	0.14	0.09	0.12	0.09	RI, MS
4	Sabinene	972	0.04	0.05	0.03	0.04	0.04	RI, MS
5	6-Methyl-5-hepten-2-one	987	0.03	0.02	0.02	0.02	0.02	RI, MS
6	*β*-Myrcene	988	0.03	0.02	0.02	0.02	0.04	RI, MS
7	*α*-Terpinene	1017	0.12	0.10	0.08	0.08	0.09	RI, MS
8	Paracymene	1024	0.02	—	—	—	—	RI, MS
9	Limonene	1027	0.71	0.63	0.36	0.5	0.39	RI, MS
10	1,8-Cineole	1031	1.97	1.44	1.66	1.41	1.97	RI, MS
11	cis-*β*-Ocimene	1037	0.02	0.02	—	0.02	0.01	RI, MS
12	trans-*β*-Ocimene	1045	0.04	0.03	0.05	0.03	0.05	RI, MS
13	*γ*-Terpinene	1054	0.33	0.13	0.15	0.18	0.14	RI, MS
14	trans-Sabinene hydrate	1096	0.29	0.14	0.17	0.17	0.13	RI, MS
15	6-Camphenol	1110	0.08	0.04	0.05	0.06	0.05	RI, MS
16	cis-Limonene oxide	1117	0.16	0.10	0.14	0.13	0.22	RI, MS
17	Campholene aldehyde	1125	0.58	0.26	0.4	0.4	0.40	RI, MS
18	trans-p-Mentha-2,8-dienol	1127	0.18	0.17	0.16	0.10	0.12	RI, MS
19	cis-p-Mentha-2,8-dien-1-ol	1131	0.33	0.18	0.20	0.21	0.15	RI, MS
20	trans-Verbenol	1144	0.64	0.34	0.47	0.38	0.45	RI, MS
21	cis-Verbenol	1153	7.78	4.87	5.22	5.82	4.41	RI, MS
22	1,3,4-Trimethyl-3-cyclohexene-1-carboxaldehyde	1171	0.70	0.63	0.59	0.53	0.62	RI, MS
23	cis-p-Mentha-1(7),8-dien-2-ol	1185	0.13	0.09	0.14	0.07	0.07	RI, MS
24	Verbenyl ethyl ether	1186	0.31	0.30	0.24	0.35	0.31	RI, MS
25	2-Carene	1189	1.71	0,86	1.11	1.03	0.84	RI, MS
26	Myrtenol	1193	0.58	0.33	0.36	0.37	0.32	RI, MS
27	trans-2-Caren-4-ol	1222	0.53	0.29	0.43	0.33	0.42	RI, MS
28	D-Carvone	1223	0.27	0.22	0.26	0.19	0.34	RI, MS
29	cis-Carveol	1226	0.06	0.03	0.04	0.05	0.03	RI, MS
30	Nerol	1228	1.85	2.16	1.56	2.05	1.60	RI, MS
31	Neral	1242	10.02	7.23	8.57	8.18	6.37	RI, MS
32	Piperitone	1250	0.15	0.11	0.14	0.12	0.07	RI, MS
33	2,6,6-Trimethyl-1-cyclohexene-1-acetaldehyde	1254	0.27	0.12	0.19	0.18	0.15	RI, MS
34	Perillic aldehyde	1257	0.03	0.03	0.02	0.03	0.04	RI, MS
35	para-Cymen-7-ol	1289	0.05	0.09	0.13	0.05	0.10	RI, MS
36	8,11,14-Eicosatrienoic acid. (Z.Z.Z)-	1294	0.04	0.05	0.03	0.03	0.03	RI, MS
37	1-(1,3-Dimethyl-buta-1.3-dienyl)-3,7,7-trimethyl-2-oxa- bicyclo[3.2.0]hept-3-ene	—	0.03	0.03	0.03	0.03	0.06	RI, MS
38	trans-Carvyl acetate	1342	0.07	0.07	0.07	0.08	0.04	RI, MS
39	Eugenol	1356	0.19	0.22	0.20	0.21	0.31	RI, MS
40	Geranyl acetate	1365	0.66	1.22	0.91	1.29	1.06	RI, MS
41	*α*-Copaene	1375	1.25	1.73	1.27	1.45	2.16	RI, MS
42	Isoledene	1377	3.48	4.46	3.89	4.19	4.04	RI, MS
43	7-Tetracyclo [6.2.1.0 (3.8)0(3.9)] Undecanol, 4,4,11,11-tetramethyl-	—	0.10	0.17	0.12	0.14	0.13	RI, MS
44	Di-epi-*α*-cedrene	1412	0.67	0.96	0.67	0.82	0.68	RI, MS
45	*β*-Caryophyllene	1420	3.11	3.26	2.77	4.18	1.85	RI, MS
46	ç-Elemene	1433	2.30	3.04	2.77	2.99	2.93	RI, MS
47	trans-*α*-Bergamotene	1434	0.09	0.09	0.08	0.09	0.04	RI, MS
48	Aromadendrene	1440	0.06	0.05	0.06	0.1	0.05	RI, MS
49	*α*-Humulen	1453	0.6	0.49	0.59	0.61	0.20	RI, MS
50	Alloaromadendrene	1460	1.66	1.36	1.02	1.30	1.00	RI, MS
51	Germacrene-D	1480	0.28	0.39	0.29	0.36	0.31	RI, MS
52	Ar-curcumene	1483	11.47	13.38	11.28	14.15	11.30	RI, MS
53	*β*-Guaiene	1490	0.01	0.01	0.02	0.02	0.01	RI, MS
54	Tricyclo[5.2.2.0(1.6)]undecan-3-ol, 2-methylene-6,8,8-trimethyl-	1498	0.49	0.68	0.56	0.56	0.65	RI, MS
55	*α*-Muurolene	1499	—	0.04	—	0.04	—	RI, MS
56	ç-Himachalene	1505	0.34	0.60	0.47	0.46	0.64	RI, MS
57	Calamenene	1514	0.09	0.16	0.11	0.12	0.13	RI, MS
58	9-Isopropyl-1-methyl-2-methylene-5-oxatricyclo[5.4.0.0(3,8)]undecane	1522	0.05	0.09	0.07	0.08	0.09	RI, MS
59	*γ*-Cadinene	1523	0.17	0.26	0.19	0.23	0.22	RI, MS
60	ç-Cadinene	1524	3.89	5.04	4.57	4.99	4.86	RI, MS
61	*β*-Spathulenol	1576	13.27	10.19	15.61	9.42	13.42	RI, MS
62	trans-Caryophyllene oxide	1580	13.52	13.25	14.14	13.28	14.22	RI, MS
63	Ledene oxide-(I)	1631	1.75	2.96	2.41	2.25	3.01	RI, MS
64	8-Cedren-13-ol	1657	—	0.02	0.03	0.01	0.05	RI, MS
65	Alloaromadendrene oxide-(1)	1672	3.06	4.26	4.37	3.79	4.35	RI, MS
66	Alloaromadendrene oxide-(2)	1678	0.28	0.50	0.4	0.4	0.79	RI, MS
67	Eudesma-4,11-dien-2-ol	1690	0.31	0.46	0.46	0.45	0.62	RI, MS
68	trans-Nuciferol	1727	2.31	3.10	2.60	3.10	3.86	RI, MS
69	cis-Nuciferol	1734	2.17	3.30	2.32	2.80	3.17	RI, MS
70	Murolan-3,9(11)-diene-10-peroxy	—	0.36	0.53	0.5	0.48	0.73	RI, MS
71	Ledene oxide-(II)	2062	0.35	0.45	0.47	0.45	0.57	RI, MS
72	Tricyclo[5.2.2.0(1.6)]undecan-3-ol, 2-methylene-6,8,8-trimethyl-	—	0.97	1.25	1.31	1.33	1.35	RI, MS

^a^Compounds are listed in order of their elution from a DB-5 column. ^b^Linear retention index taken from NIST 08, National Institute of Standards and Technology, Mass Spectral Library (NIST/EPA/NIH). ^c^Essential oil of Agadir. ^d^Essential oil of Beni Mellal. ^e^Essential oil of Berkane. ^f^Essential oil of Demnate. ^g^Essential oil of Marrakech.

**Table 2:** Main major compounds of essential oil of *Aloysia citriodora *analyzed by GC- MS.

Molecules	% AG^a^	% BM^b^	% BE^c^	% DE^d^	% MA^e^
trans-Caryophyllene oxide	13.52	13.25	14.14	13.28	14.22
*β*-Spathulenol	13.27	10.19	15.61	9.42	13.42
Ar-curcumene	11.47	13.38	11.28	14.15	11.30
Neral	10.02	7.23	8.57	8.18	6.37
cis-Verbenol	7.78	—	5.22	5.82	—

^a^AG: Agadir. ^b^BM: Beni Mellal. ^c^BE: Berkane. ^d^DE: Demnate. ^e^MA: Marrakech.

**Table 3:** Inhibition zone diameters (mm) of *A. citriodora *essential oils on bacterial strains.

Bacterial strains	Inhibition diameter (mm)						
	AG^*∗*^	BM^*∗*^	BE^*∗*^	DE^*∗*^	MA^*∗*^	Ceftriaxone	Ofloxacin
*E. coli* ATCC 25922	8	10	8	8	10	28	32
*S. aureus *ATCC 25923	8	11	11	11	11	25	28
*P. aeruginosa *ATCC 27853	6	6	6	6	6	14	18

^*∗*^AG: Agadir. BM: Beni Mellal. BE: Berkane. DE: Demnate. MA: Marrakech.

**Table 4:** Minimal inhibitory concentrations (MIC) and minimal bactericidal concentrations (MBC) of *A. citriodora *essential oils.

	MIC and MBC in mg/ml			
	*E. coli*		*S. aureus*	
	MIC	MBC	MIC	MBC
AG^*∗*^	8.37 ± 1.27	12.48 ± 2.27	5.84 ± 0.44	9.73 ± 1.81
BM^*∗*^	3.73 ± 1.00	7.42 ± 0.30	3.51 ± 0.37	9.51 ± 2.80
BE^*∗*^	5.24 ± 0.17	8.49 ± 0.33	6.42 ± 0.46	12.12 ± 1.28
DE^*∗*^	5.68 ± 1.25	9.15 ± 1.53	3.87 ± 0.36	10.31 ± 2.79
MA^*∗*^	2.84 ± 0.83	10.06 ± 3.93	4.69 ± 1.15	7.43 ± 1.32

^*∗*^AG: Agadir. BM: Beni Mellal. BE: Berkane. DE: Demnate. MA: Marrakech.
